# Satellite tag derived data from two Antarctic blue whales (*Balaenopteramusculusintermedia*) tagged in the east Antarctic sector of the Southern Ocean

**DOI:** 10.3897/BDJ.10.e94228

**Published:** 2022-12-30

**Authors:** Virginia Andrews-Goff, Elanor M Bell, Brian S Miller, Simon J Wotherspoon, Michael C Double

**Affiliations:** 1 Australian Antarctic Division, Kingston, Australia Australian Antarctic Division Kingston Australia

**Keywords:** satellite telemetry, satellite tag, Antarctic blue whale, conservation, management, foraging, Antarctica

## Abstract

**Background:**

Satellite tags were deployed on two Antarctic blue whales (*Balaenopteramusculusintermedia*) in the east Antarctic sector of the Southern Ocean as part of the International Whaling Commission’s Southern Ocean Research Partnership initiative. The satellite tracks generated are the first and currently, the only, satellite telemetry data that exist for this critically endangered species. These data provide valuable insights into the movements of Antarctic blue whales on their Antarctic feeding ground. The data were collected between February and April 2013 and span a 110° longitudinal range.

**New information:**

This dataset is the first and only detailed movement data that exist for this critically endangered species. As such, this dataset provides the first measures of movement rates (distances travelled, speeds) and movement behaviour (distinguishing transit behaviour from area restricted search behaviour) within the Southern Ocean. These movement-based measures are critical to the ongoing management of Antarctic blue whales as they recover from commercial whaling as they provide insight into foraging behaviour, habitat use, population structure and overlap with anthropogenic threats.

## Introduction

Antarctic blue whales are the largest of the blue whale subspecies. Targeted by the whaling industry during the 20^th^ century, this most numerous of the blue whale subspecies was reduced to as few as just 360 individuals (Branch et al. 2004). Protection of Antarctic blue whales by the International Whaling Commission (IWC) commenced in 1964; however, illegal Soviet whale killing continued until 1973. By this time, approximately 290,000 Antarctic blue whales were killed accounting for around 90% of the abundance and historical catches of blue whales globally (Branch et al. 2004, Branch 2008). When last assessed in 1998, the population was thought to be recovering at 7-8% per annum numbering at 2280 individuals (95% CI = [1284, 4049], CV = 0.36; Branch et al. 2004, Branch et al. 2007, Branch 2008), but is currently listed as critically endangered by the IUCN and remains protected by the IWC globally (Cooke 2018).

The little that is known of Antarctic blue whale individual movements has been constructed via the retrieval of whaling era Discovery marks from marked whales (Branch et al. 2007) and photo identification (Olson et al. 2020). These data streams relay similar, variable patterns of movement. For example, retrieval of Discovery marks have found that Antarctic blue whales sometimes disperse widely over time; however, there is no clear relationship between the distance caught from the marking location in relation to the amount of time passed since marking (Branch et al. 2007). Movements inferred from photo identification marks and recaptures have indicated that some Antarctic blue whales return to the same general area that they were initially marked (photo identified) over multiple years, whilst other whales disperse widely (Olson et al. 2020).

Discovery mark and photo identification data infer movement between two (or more) known locations at two (or more) separate points in time. The true movement path of the whale between these points in time is not known. As such, detailed movements including large scale migration between breeding and feeding grounds and fine scale movement within a feeding ground remain poorly understood.

Satellite tags are key to providing detailed, long-term movement data. Here, we present the satellite tag-derived movements of two Antarctic blue whales tagged during the austral summer in east Antarctica. These are the first and currently, the only, satellite tracks that exist for Antarctic blue whales. Deploying satellite tags on Antarctic blue whales proved to be no easy task and required the development of novel real time acoustic tracking techniques (Miller et al. 2015) and the skills and capability to closely approach fast moving blue whales within the challenging Southern Ocean environment. Even at a sample size of two, these tracks are critical to informing the ongoing management of Antarctic blue whales via the International Whaling Commission’s in-depth assessment of Antarctic blue whales due to begin in 2024 (IWC 2022) as they provide insight into population structure, distribution and movement rates, as well as occupancy of, and fidelity to, management areas or ocean basins.

## General description

### Purpose

Satellite tags were deployed during the inaugural voyage of the International Whaling Commission’s Southern Ocean Research Partnership (IWC-SORP) Antarctic Blue Whale Project (ABWP) in order to improve understanding of Antarctic blue whale population structure and movements. In particular, they were used to determine movement pathways between breeding and feeding grounds and examine whale behaviour on the feeding grounds. Satellite tags had not been deployed on Antarctic blue whales previously and proved to be logistically challenging. Antarctic blue whales were fast moving and often encountered in exposed oceanic habitat with submerged or floating ice, requiring expert navigation by the coxswain to ensure the safety of both the small boat team and the whale being sampled, hence, the small sample size. The voyage (January - March 2013) successfully employed acoustic tracking techniques to detect and locate Antarctic blue whales in real time (Miller et al. 2015). Mark-recapture data were then prioritised and collected as photo or genetic (via a biopsy sample) identification with the aim of contributing to a new Antarctic blue whale abundance estimate (Olson et al. 2021). All data are public and held by the Australian Antarctic Data Centre (https://data.aad.gov.au/).

The IWC-SORP ABWP is ongoing and represents a coordinated, international research programme focused on understanding both the recovery of Antarctic blue whales and their important role in the Southern Ocean ecosystem by employing a multi-disciplinary approach to investigate foraging ecology, habitat preferences and ultimately contributing to a precise circumpolar Antarctic blue whale abundance estimate. Since 2013, there have been around 17 voyages associated with the IWC-SORP ABWP, but no attempt has been made to deploy additional satellite tags.

### Additional information

**Figure 1.**
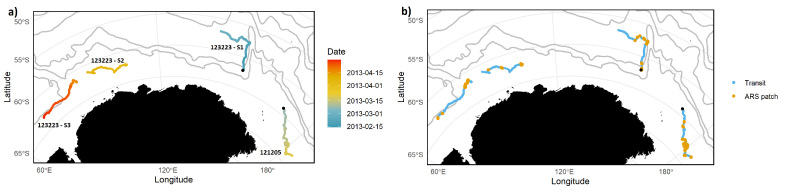
Satellite tag-derived movements of two Antarctic blue whales with Southern Ocean frontal positions (Park and Durand 2019, Park et al. 2019) in grey. From south to north, these are: Southern Boundary, Southern Antarctic Circumpolar Current, Polar, Subantarctic and Northern Boundary. **a)** State space model derived location estimates by date. Black points indicate satellite tag deployment locations. Each track is labelled by PTT and by track segment for PTT 123223. **b)** State space model derived location estimates with behavioural context – blue points indicate transit-like behaviour and orange points indicate area restricted search (ARS)-like behaviour.

The study revealed the following key results:

- The satellite tag-derived movements show contrasting movement patterns. One of the whales (PTT 123223) initially travelled north and then west for a minimum distance of 5550 km across 74 days. The other whale, PTT 121205, covered a minimum distance of 1390 km in a southeasterly direction over 13 days (Fig. 1).
- Whales moved 96 ± 43 km per day (mean ± SD); 210 km was the maximum distance travelled per day.
- Whales travelled at a speed of 4.2 ± 2.9 kmh^-1^ (median speed: 3.7 kmh^-1^; maximum speed 18.3 kmh^-1^).
- Within each track, movement indicative of transit was distinguished from movement indicative of area restricted search (ARS; putative foraging). When in ARS, swimming speed was 3.0 ± 2.1 kmh^-1^ (median: 2.5 kmh^-1^) and when in transit, swimming speed was 4.9 ± 3.1 kmh^-1^ (median 4.2 kmh^-1^).

## Project description

### Title

Satellite tag-derived data from two Antarctic blue whales (*Balaenopteramusculusintermedia*) tagged in the east Antarctic sector of the Southern Ocean as part of the IWC-SORP Antarctic Blue Whale Project.

### Personnel

Virginia Andrews-Goff, Mick Davidson, David Donnelly, Melinda Rekdahl, Natalie Schmitt (small boat team).

### Study area description

The IWC-SORP ABWP voyage occurred within the survey region bounded to the south by the 60°S parallel and between 135°E and 170°W. This survey region was identified prior to the voyage, based on an examination of catch, sightings and acoustic data that suggested the area may have a higher density of blue whales than the circumpolar average (Kelly et al. 2013). The survey employed continuous, real-time acoustic tracking to locate groups of Antarctic blue whales that were widely dispersed across this large area of Southern Ocean in order to locate whales for photographic identification and genetic sampling (Miller et al. 2015). The ship track and associated information are presented in Double et al. (2013).

The satellite-tagged Antarctic blue whales ranged widely and outside of the IWC-SORP ABWP survey region. During the tracking period, the whales traversed across an area spanning 110° in longitude and 12° in latitude, largely remaining within east Antarctica, but crossing the antemeridian to extend 4° into west Antarctica. Movement occurred across IWC Management Areas IV (70°E to 130°E; Donovan (1991)) and V (130°E to 170°W; Donovan (1991)), but remained south of the polar front and crossed the Antarctic circumpolar current. Whilst predominantly confined to the Southern Ocean (defined here as south of 60°S and including movements through the Ross Sea, Dumont d’Urville Sea and Davis Sea), movement did cross 3° into the South Pacific Ocean. The tracking period between mid-February and late April covered a time period of stable and then advancing sea ice. Sea ice retreat generally occurs November through to January and sea ice formation begins in March-April (Massom et al. 2013).

### Design description

The survey design employed during the IWC-SORP ABWP is presented in Double et al. (2013).

### Funding

The inaugural Antarctic Blue Whale Voyage (2013) was funded by the Australian Government’s International Whale and Marine Mammal Conservation Initiative (IWMMCI).

## Sampling methods

### Study extent

Whales moved broadly through the east Antarctic sector of the Southern Ocean.

### Sampling description

**Satellite tag deployment**

Type C implantable satellite tags (Andrews et al. 2019) were deployed on two adult Antarctic blue whales with a modified version of the Air Rocket Transmitter System (ARTS), Restech (Heide‐Jørgensen et al. 2001) and a purpose-designed projectile carrier at a pressure of 7.5 – 8.5 bar. The satellite tag employed was comprised of an 80 mm anchor section attached to a stainless steel cylindrical housing containing a location-only Spot 200 transmitter manufactured by Wildlife Computers (Redmond, Washington, USA). These tags were fitted with a stainless-steel collar to reinforce the bolt that connects the anchor to the cylindrical electronics housing; however, this design is now superseded. Retention teeth on a purpose-designed projectile carrier grip a metal ring fitted to the end of the tag allowing the tag to be fired from the ARTS. When the tag makes contact with the whale, the rapid deceleration of the tag and the projectile carrier withdraws the retention teeth releasing the projectile carrier. The metal ring then falls off in time to reduce the drag of the tag. The tag was sterilised with ethylene oxide prior to deployment and implants up to a maximum of 290 mm into the skin, blubber, interfacial layers and outer muscle mass of the whale. Two actively sprung plates and a circle of passively deployed ‘petals’ aid tag retention.

Each tag was deployed from the bow-sprit of a purpose-built 6.3 m aluminium Naiad RHIB and was positioned high and forward on the body, approximately in line with the pectoral fins. When the tag is immersed in salt water, the salt water switch activates and the tag begins to transmit locations via the Argos satellite system. Tags were programmed to transmit to the Argos satellite system at a duty cycle of three hour on/three hour off and a 30 second repetition rate to extend battery life. These transmissions are relayed to processing centres which calculate the transmitter’s location by measuring the Doppler Effect on transmission frequency. Transmitted data were processed using a least squares analysis and each location was assigned an estimated error and one of seven associated location classes (see CLS 2016). Tags cease transmitting when they are either naturally shed, undergo damage, undergo sensor fouling or the battery is exhausted.

Upon tag deployment, a small amount of skin and blubber was simultaneously collected for genetic analyses. These were collected using a biopsy dart fired from a modified 0.22 Paxarms system (Krutzen et al. 2002). Biopsy samples were stored in 70% ethanol and DNA subsequently extracted using a Tissue DNA purification kit for the Maxwell 16 DNA extraction robot (Promega Corporation). The sexes of the tagged whales were determined using a 5′ exonuclease assay of the polymorphisms in the sex-linked Zinc Finger genes as described by Morin et al. (2005). Photo-identification images were also collected simultaneously with tag deployment. Photographs from the tagged whales were compared to those in the Antarctic Blue Whale Catalogue (Olson et al. 2013).

This research was conducted using non-lethal methods that are designed to learn about whales without harming them. The research was approved by the Australian Antarctic Ethics Committee (under Australian Antarctic Science Project 4102) and complied with all relevant permits including the Australian Government Environment Protection and Biodiversity Conservation Act Cetacean Permit (C12-0006).

### Quality control

**Argos data processing**

Argos locations were filtered using an algorithm, based on swimming speed, distance between successive locations and turning angles using the using the R (R Core Team 2021) package *Argosfilter* (Freitas et al. 2008) to remove unlikely position estimates (speed of 10 ms^−1^, spike angles of 15° and 25°, spike lengths of 2500 m and 5000 m). Removals were verified manually via visual inspection. This resulted in the removal of 15% (PTT 123223) and 22% (PTT 121205) of locations for each whale, respectively (Table 1).

**Argos location error**

To account for the spatial error associated with Argos locations, we fit a random walk state-space model to estimate locations at a two hour time step (*fit_ssm* function in the *foieGras* package; Jonsen et al. (2019)). Individual tracks were split into track segments where data gaps exceeded 24 hours. The state-space-model was implemented per track segment > 10 locations resulting in the removal of two short track segments (n = 5 locations over 12 days at the end of PTT 123223 track segment 1 and n = 6 locations over 1 day for PTT 123223 at the start of track segment 2; see also Temporal coverage for retained track segment details).

**Behavioural context**

In order to provide context to the observed movement, a move persistence model was fitted to the state-space location estimates of each track (*fit_mpm* function in *foieGras* with unpooled random walk variance parameters; Jonsen et al. (2019)). The move persistence model assigns a behavioural classification to each state-space location estimate. The move persistence model estimates the time-varying autocorrelation in speed and directionality along the track generating a move persistence value (gamma) at each location. Move persistence ranges along a continuum between 0 and 1 - move persistence values approaching 1 indicate directed travel (transit) and move persistence values approaching 0 represent slower, tortuous movements (area restricted search - ARS), representative of putative foraging. We used the mean of all gamma values as a cut-off point to categorically assign each location as either transit or ARS. Following Bailey et al. (2009) and Andrews-Goff et al. (2018), we assigned ARS patches, comprising of successive location estimates classified as ARS, ending when three or more consecutive location estimates are classified as transit. These patches represent distinct clusters of area restricted search.

## Geographic coverage

### Description

The geographic range of the bulk of the dataset is within the east Antarctic sector of the Southern Ocean, south of the polar front and crossing the Antarctic circumpolar current (Fig. 1).

### Coordinates

-68.9 and -57. Latitude; 184.4 and 73.9 Longitude.

## Taxonomic coverage

### Description

This dataset focuses exclusively on the Antarctic blue whale (*Balaenopteramusculusintermedia*), which is categorised as Critically Endangered in the IUCN Red List (Cooke 2018). It belongs to the family Balaenopteridae within the order Cetartiodactyla.

### Taxa included

**Table:** 

Rank	Scientific Name	
kingdom	Animalia	
phylum	Chordata	
class	Mammalia	
order	Cetartiodactyla	
family	Balaenopteridae	
genus	Balaenoptera	
species	*Balaenopteramusculusintermedia*	Antarctic blue whale

## Temporal coverage

**Data range:** 2013-2-14 – 2013-4-29.

### Notes

The transmission period for PTT 121205 was continuous, spanning 13 days, date range: 08/03/2013 to 21/03/2013. The transmission period for PTT 123223 was not continuous. The entire track for PTT 123223 can be seen in Fig. 1 as three track segments:

Track segment 1: 14/02/2013 to 01/03/2013, 13 days

Track segment 2: 01/04/2013 to 08/04/2013, 7 days

Track segment 3: 16/04/2013 to 29/04/2013, 13 days

## Usage licence

### Usage licence

Other

### IP rights notes

CC BY: This licence allows reusers to distribute, remix, adapt and build upon the material in any medium or format, so long as attribution is given to the creator. The licence allows for commercial use.

## Data resources

### Data package title

Antarctic blue whale tracking data - satellite tag-derived Argos locations and associated information, state space model with move persistence/behavioural index and the reference data detailing the satellite tag deployments. These datasets are published on Movebank (https://www.movebank.org/cms/webapp?gwt_fragment=page=studies,path=study2391441038), GBIF (https://www.gbif.org/dataset/6942e235-3ac0-418a-a042-f515cc7da235) and the Australian Antarctic Data Centre (https://data.aad.gov.au/metadata/AAS_4102_sat_tag).

### Resource link

https://www.movebank.org/cms/webapp?gwt_fragment=page=studies,path=study2391441038

### Number of data sets

3

### Data set 1.

#### Data set name

**Antarctic blue whales east Antarctic sector of the Southern Ocean**

#### Data format

CSV file

#### Download URL

https:// doi: 10.5441/001/1.vr276ns3

#### Description

This file contains all Argos locations generated by the two satellite tags.

**Data set 1.** 

Column label	Column description
event ID	An identifier for the set of values associated with each event. A unique event ID is assigned to every time-location record.
visible	Determines whether an event is visible on the Movebank map.
timestamp	The date and time corresponding to each location estimate. Format: yyyy-MM-dd HH:mm:ss.SSS; units: UTC.
location long	The geographic longitude of the location as estimated by the sensor. Positive values are east of the Greenwich Meridian, negative values are west of it. Units: decimal degrees, WGS84 reference system.
location lat	The geographic longitude of the location as estimated by the sensor. Units: decimal degrees, WGS84 reference system.
argos:calcul-freq	Calculated frequency, Argos diagnostic data. It should be between 401.620 and 401.680 MHz (definition from Argos User's Manual 2011). The '401.' is sometimes missing from the source data and should be added to the values for correct intepretation.
argos:iq	This quality indicator gives information on the transmitter in terms of two digits, X and Y. X is the first digit and indicates residual error on the frequency calculation; Y is the second digit and indicates transmitter oscillator frequency drift between two satellite passes. Values provided in Argos diagnostic data (definition from Argos User's Manual 2011). Values obtained through some Argos channels do not include leading 0s, so 1-digit values indicate X = 0 and blank values or values of '0' indicate both X and Y = 0. Allowed values are X = 0: No calculation of residual frequency error (fewer than four messages received); X = 1,2,3: Unsatisfactory convergence of calculation; X = 4: Residual frequency error > 1.5 Hz; X = 5: 0.15 Hz < residual frequency error < 1.5 Hz; X = 6: Residual frequency error < 0.15 Hz; Y = 0: No check on transmit frequency drift, as the two results are more than 12 hours apart; Y = 1: Frequency discrepancy > 400 Hz; Probably due to transmit frequency discrepancy, change of oscillator etc.; Y = 2: Previous location is less than 1/2 hour old. Frequency discrepancy > 30 Hz, i.e. F/F (over 10 min) > 2.5 E-8; Y = 3: Frequency drift > 4 Hz/minute, i.e. F/F (10 min) > 1.10-7; Y = 4: Frequency drift < 4 Hz/minute, i.e. F/F (10 min) < 1.10-7; Y = 5: Frequency drift < 2 Hz/minute, i.e. F/F (10 min) < 5.10-8; Y = 6: Frequency drift < 1 Hz/minute, i.e. F/F (10 min) < 2.5 . 10-8; Y = 7: Frequency drift < 0.4 Hz/minute, i.e. F/F (10 min) < 1.10-8; Y = 8: Frequency drift < 0.2 Hz/minute, i.e. F/F (10 min) < 5.10-9.
argos:lc	Argos LC: The location class retrieved from Argos, Argos diagnostic data. Classes are based on the type of location (Argos Doppler Shift or GPS) and the number of messages received during the satellite pass. Location classes in order of decreasing accuracy are G (GPS), 3, 2, 1, 0, A, B and Z (definition from Argos User's Manual V1.6.6, 2016).
argos:location-algorithm	The processing algorithm used by Argos to estimate locations using Doppler shift. If the location data represent model output rather than the original estimates from Argos, also use 'modelled'. Values are chosen from a controlled list: least squares = locations were calculated by Argos using a least-squares analysis; Kalman = locations were calculated by Argos using Kalman filtering.
argos:nb-mes	The number of messages received [to calculate location], Argos diagnostic data (definition from Argos User's Manual 2011).
argos:sat-id	The satellite identifier, Argos diagnostic data (definition from Argos User's Manual 2011).
sensor-type	The type of sensor with which data were collected. Argos Doppler shift = The sensor location is estimated by Argos using Doppler shift.
individual-taxon-canonical-name	The scientific name of the species on which the tag was deployed, as defined by the Integrated Taxonomic Information System (ITIS, www.itis.gov).
tag-local-identifier	An identifier for the tag.
individual-local-identifier	An individual identifier for the animal.
study-name	The name of the study in Movebank.

### Data set 2.

#### Data set name

BDJ_ssm_2h_mpm

#### Data format

CSV file

#### Download URL

https:// doi: 10.5441/001/1.vr276ns3

#### Description

The random walk state-space model output used to account for Argos location error with an estimated location every 2 hours. This state-space model was used as the input for the move persistence model and the behavioural index (gamma) is included here.

**Data set 2.** 

Column label	Column description
id	The individual identifier for each track segment - the first 6 digits are equal to PTT which is followed by an underscore and then a number to indicate the unique track segment belonging to that PTT.
date	Date (yyyy-mm-dd hh:mm:ss) in UTC.
lon	State space model predicted longitude in decimal degrees.
lat	State space model predicted latitude in decimal degrees.
g	Gamma value used as the behavioural index.
mode	The assigned behavioural mode - ARS is assigned where g < mean(g) and transit is assigned where g >= mean(g).
patch	Assigning each location to an ARS patch - FALSE indicates outside of an ARS patch, TRUE indicates within an ARS patch.

### Data set 3.

#### Data set name

Antarctic blue whales east Antarctic sector of the Southern Ocean-reference-data

#### Data format

CSV

#### Download URL

https:// doi: 10.5441/001/1.vr276ns3

#### Description

Reference data detailing satellite tag deployments on Antarctic blue whales.

**Data set 3.** 

Column label	Column description
tag-id	A unique identifier for the deployment of a tag on animal.
animal-id	An individual identifier for the animal.
animal-taxon	The scientific name of the species on which the tag was deployed, as defined by the Integrated Taxonomic Information System (ITIS, www.itis.gov).
deploy-on-date	The timestamp when the tag deployment started. Format: yyyy-MM-dd HH:mm:ss.SSS units: UTC.
deploy-off-date	The timestamp when the tag deployment ended. Format: yyyy-MM-dd HH:mm:ss.SSS units: UTC.
animal-comments	Additional information about the animal that is not described by other reference data terms.
animal-life-stage	The age class or life stage of the animal at the beginning of the deployment. Can be years or months of age or terms such as 'adult', 'subadult' and 'juvenile'.
animal-sex	The sex of the animal. Allowed values are m = male; f = female; u = unknown.
animal-taxon-detail	The scientific name of the species on which the tag was deployed, as defined by the Integrated Taxonomic Information System (ITIS, www.itis.gov).
attachment-type	The way a tag is attached to an animal; implant = the tag is placed under the skin of the animal.
deploy-on-latitude	The geographic latitude of the location where the animal was released. Units: decimal degrees, WGS84 reference system.
deploy-on-longitude	The geographic longitude of the location where the animal was released. Units: decimal degrees, WGS84 reference system.
deployment-comments	Additional information about the tag deployment that is not described by other reference data terms.
deployment-end-type	A categorical classification describing the end of the tag deployment on the animal. Unknown = The cause of the end of data availability or transmission is unknown.
deployment-id	A unique identifier for the deployment of a tag on animal.
duty-cycle	Remarks associated with the duty cycle of a tag during the deployment, describing the times it is on/off and the frequency at which it transmits or records data.
manipulation-type	The way in which the animal was manipulated during the deployment. None = The animal received no treatment other than tag attachment and related measurements and sampling.
study-site	Location of the deployment site.
tag-comments	Additional information about the tag that is not described by other reference data terms.
tag-manufacturer-name	The company or person that produced the tag.
tag-model	The model of the tag.
tag-readout-method	The way the data are received from the tag; satellite = data are transferred via satellite.

## Figures and Tables

**Table 1.** Satellite tag deployment details.

PTT	Date deployed	Last location	Location	Latitude	Longitude	Sex	ARTS pressure (bar)	Deployment distance (m)	Number of locations	Number of locations post SDA filter
123223	14/02/2013	29/04/2013	Western Ross Sea	-62.0059	149.0136	Female	7.5	5	499	426
121205	08/03/2013	21/03/2013	Western Ross Sea	-64.0408	168.2875	Male	8.5	8	319	250
